# First experience in Indonesia: early report of fifteen cases on congenital beating heart surgery with continuous coronary artery perfusion without aortic cross clamp

**DOI:** 10.1186/1749-8090-8-S1-P143

**Published:** 2013-09-11

**Authors:** PL Tahalele, Puruhito, A Prasmono, H Kusbijanto, H Soebroto, YE Sembiring

**Affiliations:** 1 Department of Surgery Division of Cardiothoracic and Vascular Surgery School of Medicine Airlangga University - Dr. Soetomo General Hospital Surabaya, Indonesia

## Background

The development of cardioplegia solution has a beneficial of clear operating view for the surgeon. Although it’s remain the standard of open heart surgery, the main concern of surgical teams has been finding the best means of protecting the heart from the harmful effects of myocardial ischemia.Over 50 years, many therapeutic strategies have been developed to protect the heart during surgery. The most renowned method is Aortic cross clamped followed by cardioplegic solutions to arrest the heart. On the other hand, there was no studies have addressed the efficacy and safety of on-pump beating-heart surgery with continuous coronary artery perfusion in Indonesia. The purpose of this study was to report the efficacy and safety of on-pump beating-heart surgery with continuous coronary artery perfusion in 15 patients with congenital cardiac disease.

## Methods

Preoperative risk factors, intraoperative techniques, and postoperative complications were documented and evaluated in 15 consecutive patients (from December 1st, 2011, to May 31st, 2013) who underwent beating-heart surgery at dr Soetomo teaching hospital in Surabaya, Indonesia. The cases varies from ASD (9) and VSD (1) closure, Total repair of TOF (2), ASD and TAPVD (2). The overall observational period for all patients were 3 months after surgery.

## Results

There were no low cardiac output syndrome occurred. No air embolization or permanent high degree atrioventricular block occurred in these patients. There was only one residual VSD (2mm) from TOF repair. The crude mortality of the surveyed patients was 0%.

## Conclusion

Our early results indicate that on-pump beating-heart surgery with continuous coronary artery perfusion is safe and have less effect in myocardial stunning, intra operative ischemic period thus have superiority in myocardial protection.

